# Supplementary material for: HIVprotI: an integrated web based platform for prediction and design of HIV proteins inhibitors
Source: J Cheminform. 2018 Mar 9;10:12. doi: 10.1186/s13321-018-0266-y (PMC5845081; doi:10.1186/s13321-018-0266-y)
Supplement: Supplementary file 2 — Additional file 2. Source code of HIVProtI web server. [file 13321_2018_266_MOESM2_ESM.zip › HIVprotI_Source-code/hivprotI/weka-3-6-6/doc/weka/associations/tertius/Rule.html]

Rule


---


|  |  |  |
| --- | --- | --- |
| **Overview** \| **Package** \| **Class** \| **Tree** \| **Deprecated** \| **Index** \| **Help** \| **Weka's home** | |  |
| **PREV CLASS**   **NEXT CLASS** | **FRAMES**    **NO FRAMES**     **All Classes** |  |
| SUMMARY: NESTED \| FIELD \| CONSTR \| METHOD | DETAIL: FIELD \| CONSTR \| METHOD |  |


---


## weka.associations.tertius Class Rule

```
java.lang.Object
  weka.associations.tertius.Rule
```

**All Implemented Interfaces:**: java.io.Serializable, java.lang.Cloneable, RevisionHandler

---

``` public class Rule extends java.lang.Object implements java.io.Serializable, java.lang.Cloneable, RevisionHandler ```

Class representing a rule with a body and a head.

**Version:**
:   $Revision: 1.7 $

**Author:**
:   Amelie Deltour

**See Also:**: Serialized Form

---

| **Field Summary** | |
| --- | --- |
| `static java.util.Comparator` | `confirmationComparator`             Comparator used to compare two rules according to their confirmation value. |
| `static java.util.Comparator` | `confirmationThenObservedComparator`             Comparator used to compare two rules according to their confirmation and then their observed number of counter-instances. |
| `static java.util.Comparator` | `observedComparator`             Comparator used to compare two rules according to their observed number of counter-instances. |
| `static java.util.Comparator` | `optimisticComparator`             Comparator used to compare two rules according to their optimistic estimate. |
| `static java.util.Comparator` | `optimisticThenObservedComparator`             Comparator used to compare two rules according to their optimistic estimate and then their observed number of counter-instances. |


**Constructor Summary**

`Rule(boolean repeatPredicate,
int maxLiterals,
boolean negBody,
boolean negHead,
boolean classRule,
boolean horn)`
  
          Constructor for a rule when the counter-instances are not stored,
giving all the constraints applied to this rule.

`Rule(Instances instances,
boolean repeatPredicate,
int maxLiterals,
boolean negBody,
boolean negHead,
boolean classRule,
boolean horn)`
  
          Constructor for a rule when the counter-instances are stored,
giving all the constraints applied to this rule.


| **Method Summary** | |
| --- | --- |
| `boolean` | `bodyContains(Literal lit)`             Test if the body of the rule contains a literal. |
| `void` | `calculateConfirmation()`             Calculate the confirmation of this rule. |
| `void` | `calculateOptimistic()`             Calculate the optimistic estimate of this rule. |
| `java.lang.Object` | `clone()`             Returns a shallow copy of this rule. |
| `boolean` | `counterInstance(Instance instance)`             Test if an instance is a counter-instance of this rule. |
| `boolean` | `equivalentTo(Rule otherRule)`             Test if this rule is equivalent to another rule. |
| `double` | `getConfirmation()`             Get the confirmation value of this rule. |
| `double` | `getExpectedFrequency()`             Get the expected frequency of counter-instances of this rule. |
| `double` | `getExpectedNumber()` |
| `double` | `getFPRate()`             Get the rate of False Positive instances of this rule. |
| `double` | `getObservedFrequency()`             Get the observed frequency of counter-instances of this rule in the dataset. |
| `int` | `getObservedNumber()`             Get the observed number of counter-instances of this rule in the dataset. |
| `double` | `getOptimistic()`             Get the optimistic estimate of the confirmation obtained by refining this rule. |
| `java.lang.String` | `getRevision()`             Returns the revision string. |
| `double` | `getTPRate()`             Get the rate of True Positive instances of this rule. |
| `boolean` | `hasFalseHead()`             Test if the head of the rule is false. |
| `boolean` | `hasTrueBody()`             Test if the body of the rule is true. |
| `boolean` | `headContains(Literal lit)`             Test if the head of the rule contains a literal. |
| `boolean` | `isEmpty()`             Test if this rule is empty. |
| `int` | `numLiterals()`             Give the number of literals in this rule. |
| `boolean` | `overFrequencyThreshold(double minFrequency)`             Test if this rule is over the frequency threshold. |
| `SimpleLinkedList` | `refine(java.util.ArrayList predicates)`             Refine a rule by adding literal from a set of predictes. |
| `java.lang.String` | `rocToString()`             Return a String giving the TP-rate and FP-rate of this rule. |
| `boolean` | `sameClauseAs(Rule otherRule)`             Test if this rule and another rule correspond to the same clause. |
| `boolean` | `subsumes(Rule otherRule)`             Test if this rule subsumes another rule. |
| `java.lang.String` | `toString()`             Retrun a String for this rule. |
| `void` | `upDate(Instances instances)`             Update the number of counter-instances of this rule in the dataset. |
| `java.lang.String` | `valuesToString()`             Return a String giving the confirmation and optimistic estimate of this rule. |

**Methods inherited from class java.lang.Object**

`equals, getClass, hashCode, notify, notifyAll, wait, wait, wait`

**Field Detail**

### confirmationComparator

```
public static java.util.Comparator confirmationComparator
```

:   Comparator used to compare two rules according to their confirmation value.

---


### observedComparator

```
public static java.util.Comparator observedComparator
```

:   Comparator used to compare two rules according to their observed number
    of counter-instances.

---


### optimisticComparator

```
public static java.util.Comparator optimisticComparator
```

:   Comparator used to compare two rules according to their optimistic estimate.

---


### confirmationThenObservedComparator

```
public static java.util.Comparator confirmationThenObservedComparator
```

:   Comparator used to compare two rules according to their confirmation and
    then their observed number of counter-instances.

---


### optimisticThenObservedComparator

```
public static java.util.Comparator optimisticThenObservedComparator
```

:   Comparator used to compare two rules according to their optimistic estimate
    and then their observed number of counter-instances.


**Constructor Detail**

### Rule

```
public Rule(boolean repeatPredicate,
            int maxLiterals,
            boolean negBody,
            boolean negHead,
            boolean classRule,
            boolean horn)
```

:   Constructor for a rule when the counter-instances are not stored,
    giving all the constraints applied to this rule.

    **Parameters:**: `repeatPredicate` - True if predicates can be repeated.: `maxLiterals` - Maximum number of literals.: `negBody` - True if negation is allowed in the body.: `negHead` - True if negation is allowed in the head.: `classRule` - True if the rule is a classification rule.: `horn` - True if the rule is a horn clause.

---


### Rule

```
public Rule(Instances instances,
            boolean repeatPredicate,
            int maxLiterals,
            boolean negBody,
            boolean negHead,
            boolean classRule,
            boolean horn)
```

:   Constructor for a rule when the counter-instances are stored,
    giving all the constraints applied to this rule.
    The counter-instances are initialized to all the instances in the dataset.

    **Parameters:**: `instances` - The dataset.: `repeatPredicate` - True if predicates can be repeated.: `maxLiterals` - Maximum number of literals.: `negBody` - True if negation is allowed in the body.: `negHead` - True if negation is allowed in the head.: `classRule` - True if the rule is a classification rule.: `horn` - True if the rule is a horn clause.


**Method Detail**

### clone

```
public java.lang.Object clone()
```

:   Returns a shallow copy of this rule.
    The structured is copied but the literals themselves are not copied.

    :   **Overrides:**: `clone` in class `java.lang.Object`
    :   **Returns:**: A copy of this Rule.

---


### counterInstance

```
public boolean counterInstance(Instance instance)
```

:   Test if an instance is a counter-instance of this rule.

    :   **Parameters:**: `instance` - The instance to test. **Returns:**: True if the instance is a counter-instance.

---


### upDate

```
public void upDate(Instances instances)
```

:   Update the number of counter-instances of this rule in the dataset.
    This method should be used is the rule does not store its counter-instances.

    :   **Parameters:**: `instances` - The dataset.

---


### getConfirmation

```
public double getConfirmation()
```

:   Get the confirmation value of this rule.

    :   **Returns:**: The confirmation.

---


### getOptimistic

```
public double getOptimistic()
```

:   Get the optimistic estimate of the confirmation obtained by refining
    this rule.

    :   **Returns:**: The optimistic estimate.

---


### getExpectedNumber

```
public double getExpectedNumber()
```

---


### getExpectedFrequency

```
public double getExpectedFrequency()
```

:   Get the expected frequency of counter-instances of this rule.

    :   **Returns:**: The expected frequency of counter-instances.

---


### getObservedNumber

```
public int getObservedNumber()
```

:   Get the observed number of counter-instances of this rule in the dataset.

    :   **Returns:**: The observed number of counter-instances.

---


### getObservedFrequency

```
public double getObservedFrequency()
```

:   Get the observed frequency of counter-instances of this rule in the dataset.

    :   **Returns:**: The expected frequency of counter-instances.

---


### getTPRate

```
public double getTPRate()
```

:   Get the rate of True Positive instances of this rule.

    :   **Returns:**: The TP-rate.

---


### getFPRate

```
public double getFPRate()
```

:   Get the rate of False Positive instances of this rule.

    :   **Returns:**: The FP-rate.

---


### calculateConfirmation

```
public void calculateConfirmation()
```

:   Calculate the confirmation of this rule.

---


### calculateOptimistic

```
public void calculateOptimistic()
```

:   Calculate the optimistic estimate of this rule.

---


### isEmpty

```
public boolean isEmpty()
```

:   Test if this rule is empty.

    :   **Returns:**: True if it is the empty rule.

---


### numLiterals

```
public int numLiterals()
```

:   Give the number of literals in this rule.

    :   **Returns:**: The number of literals.

---


### refine

```
public SimpleLinkedList refine(java.util.ArrayList predicates)
```

:   Refine a rule by adding literal from a set of predictes.

    :   **Parameters:**: `predicates` - The predicates available. **Returns:**: The list of the children obtained by refining the rule.

---


### subsumes

```
public boolean subsumes(Rule otherRule)
```

:   Test if this rule subsumes another rule.

    :   **Parameters:**: `otherRule` - The other rule. **Returns:**: True if the other rule is subsumed.

---


### sameClauseAs

```
public boolean sameClauseAs(Rule otherRule)
```

:   Test if this rule and another rule correspond to the same clause.

    :   **Parameters:**: `otherRule` - The other rule. **Returns:**: True if both rules correspond to the same clause.

---


### equivalentTo

```
public boolean equivalentTo(Rule otherRule)
```

:   Test if this rule is equivalent to another rule.

    :   **Parameters:**: `otherRule` - The other rule. **Returns:**: True if both rules are equivalent.

---


### bodyContains

```
public boolean bodyContains(Literal lit)
```

:   Test if the body of the rule contains a literal.

    :   **Parameters:**: `lit` - The literal to look for. **Returns:**: True if the literal is contained in the body of the rule.

---


### headContains

```
public boolean headContains(Literal lit)
```

:   Test if the head of the rule contains a literal.

    :   **Parameters:**: `lit` - The literal to look for. **Returns:**: True if the literal is contained in the head of the rule.

---


### overFrequencyThreshold

```
public boolean overFrequencyThreshold(double minFrequency)
```

:   Test if this rule is over the frequency threshold.

    :   **Parameters:**: `minFrequency` - The frequency threshold. **Returns:**: True if the rule is over the threshold.

---


### hasTrueBody

```
public boolean hasTrueBody()
```

:   Test if the body of the rule is true.

    :   **Returns:**: True if the body is always satisfied.

---


### hasFalseHead

```
public boolean hasFalseHead()
```

:   Test if the head of the rule is false.

    :   **Returns:**: True if the body is never satisfied.

---


### valuesToString

```
public java.lang.String valuesToString()
```

:   Return a String giving the confirmation and optimistic estimate of
    this rule.

    :   **Returns:**: A String with the values of the rule.

---


### rocToString

```
public java.lang.String rocToString()
```

:   Return a String giving the TP-rate and FP-rate of
    this rule.

    :   **Returns:**: A String with the values of the rule.

---


### toString

```
public java.lang.String toString()
```

:   Retrun a String for this rule.

    :   **Overrides:**: `toString` in class `java.lang.Object`
    :   **Returns:**: The String describing this rule.

---


### getRevision

```
public java.lang.String getRevision()
```

:   Returns the revision string.

    :   **Specified by:**: `getRevision` in interface `RevisionHandler`
    :   **Returns:**: the revision


---


|  |  |  |
| --- | --- | --- |
| **Overview** \| **Package** \| **Class** \| **Tree** \| **Deprecated** \| **Index** \| **Help** \| **Weka's home** | |  |
| **PREV CLASS**   **NEXT CLASS** | **FRAMES**    **NO FRAMES**     **All Classes** |  |
| SUMMARY: NESTED \| FIELD \| CONSTR \| METHOD | DETAIL: FIELD \| CONSTR \| METHOD |  |


---
